# Unmet need for family planning and predictors among women in the extended postpartum period, southeastern Nigeria: a facility-based cross-sectional study

**DOI:** 10.11604/pamj.2023.45.38.39205

**Published:** 2023-05-16

**Authors:** Chidinma Ihuoma Amuzie, Kalu Ulu Kalu, Michael Izuka, Glory Emeka Nkwo, Uche Ngozi Nwamoh, Kingsley Metu, Uloaku Emma-Ukaegbu, Godwin Oguejiofor Okafor, Franklin Odini

**Affiliations:** 1 Department of Community Medicine, Federal Medical Centre, Umuahia, Abia State, Nigeria,; 2 Nigeria Field Epidemiology and Laboratory Training Program, Abuja, Nigeria

**Keywords:** Contraception, family planning, maternal health, postpartum period, postpartum women, Nigeria

## Abstract

**Introduction:**

the ravaging COVID-19 pandemic has worsened the levels of unmet need (UMN) for family planning (FP). A pulse survey showed that FP services were interrupted during the lockdown in 68% of countries. There is a need to investigate the demand gap for FP among women in the postpartum period. This study aimed to determine the prevalence and predictors of UMN of FP among women in the extended postpartum period attending immunization clinics.

**Methods:**

this was a facility-based cross-sectional study among 485 women recruited from 5 health facilities using a random sampling technique and proportional to size allocation. Data was collected using an interviewer-structured questionnaire. Analysis was done using IBM SPSS version 26. Adjusted odds ratios with 95% confidence intervals were computed. The level of significance was 5%.

**Results:**

the total unmet need was 45.4% (95% CI: 40.2–50.7) with a higher need for child spacing (60.4%) compared to limiting pregnancies (39.6%). The mean age was 30.3 ± 6.1 years. The significant predictors included age [35-39 years (aOR=5.39, 95% CI: 1.61-18.06); ≥ 40 years (aOR= 32.48, 95% CI: 6.48-162.77)], lower education status (aOR= 5.21, 95% CI: 2.09-13.01), lower income (aOR =2.21, 95% CI: 1.10-4.46), rural residence (aOR= 4.27, 95% CI: 2.15-8.47), denomination [Pentecostal (aOR= 4.09, 95% CI: 1.77-9.43); Orthodox (aOR= 5.44, 95% CI: 2.03-14.58)] and poor knowledge of postpartum FP (PPFP) (aOR= 33.93, 95% CI: 13.21-87.12). The commonest reason for FP non-use was fear of side effects.

**Conclusion:**

close to half of the women in the extended postpartum period experienced UMN for PPFP. Policymakers should consider these factors when designing FP interventions.

## Introduction

There is a growing concern about the impact of the COVID-19 pandemic on essential health services [1]. Many countries in Africa, including Nigeria, have been grossly affected by the pandemic, and the fear of worsening maternal health indicators is heightened [2]. During the lockdown, there was a global disruption in the supply of contraceptives [3]. With the growing pandemic and succession in waves, women are likely to experience some hindrances to sexual and reproductive health care services, including PPFP services [3]. Restrictions have been eased in several countries, but the effects on the health system are still palpable.

According to the World Health Organization (WHO), women with UMN are defined as those who are fecund and sexually active but are not using any method of contraception and report not wanting any more children or wanting to delay the next child [4]. Postpartum family planning is defined as the prevention of unintended pregnancy and closely spaced pregnancies through the first 12 months following childbirth [5]. The utilization of contraceptives during this period is known to be cost-effective as it is integrated with child health services [5]. A pulse survey conducted by WHO showed that FP services were interrupted during the lockdown in 68% of countries, with 9% of those countries reporting severe or complete disruption [6]. Over the past two decades, the number of women desiring to use FP has drastically increased from 900 million in 2000 to 1.1 billion in 2020 [7].

Unmet family planning needs are prevalent in low- and middle-income countries (LMICs), with a higher burden of UMN in four countries, primarily Nigeria, Uganda, the Democratic Republic of the Congo (DRC), and Kenya [8]. This is associated with a marked increase in the total cost of care to meet all women's reproductive needs [8]. The burden of UMN has contributed to poor maternal health indices [9]. In developing countries, women with UMN of FP account for 89 million unwanted pregnancies annually [10]. This increases the likelihood of unsafe abortions and other health consequences [8]. Due to UMN, poorly spaced and ill-timed pregnancies affect the outcomes of the fetus, contributing to high infant mortality rates [11-13]. The high prevalence of UMN has also led to non-attainment of SDG target 5.6, which supports universal access to sexual and reproductive rights and the target of FP 2020 London summit. According to the 2018 National Demographic Health Survey (NDHS) in Nigeria, the total fertility rate (TFR) is 5.3 births and the expected value of TFR is 2.1 births [14]. It has been documented that a high fertility rate in women is associated with a negative impact on the economic growth of the nation [15].

Based on the literature reviewed, some predictors of UMN among postpartum women include age, women's education, rural residence, low perception of pregnancy, husband's approval and spousal communication about contraceptives; reproductive factors including parity, prior use of contraceptives, antenatal (ANC) and postnatal (PNC) services, assisted deliveries, resumption of menstruation and sexual activities and level of awareness of PPFP [16-21]. There are existing program deficiencies in FP policies addressing the postpartum FP needs of women [5]. Several studies have documented the prevalence and determinants of unmet needs among women of childbearing age. However, there is a need to provide more insights into the increasing evidence of UMN among women in the postpartum period, particularly in sub-Saharan African countries such as Nigeria [22]. Findings from this study will help improve current FP policies and practices. This study assessed the prevalence and predictors of unmet need for FP among women in the extended postpartum period.

## Methods

**Study design and setting:** a facility-based cross-sectional study was conducted among women within the extended postpartum period in Umuahia from September to October 2022. Umuahia is the capital city of Abia State in southeastern Nigeria. Umuahia North had an estimated population of 303,787 in 2018, projected from the national census of 2006. The town has two tertiary health institutions, one secondary health facility and 43 primary health care facilities that offer FP and immunization services at subsidized rates. There are 62 private health facilities in the LGA and very few offer immunization and FP services. Most of the facilities run active immunization clinics about two times a week. Health education, including FP counseling, is part of the immunization program activities. The inhabitants are mainly Christians. The Catholic denomination which is predominant in the area is known to have existing religious beliefs on FP. There are no known taboos against the uptake of FP. However, some myths concerning FP are known to exist in some communities.

**Study participants:** this included women in the extended postpartum period attending immunization clinics in the public health facilities within Umuahia North LGA, Abia State. Women of reproductive age (15-49 years) in a marital or sexual union were eligible for the study. While caregivers or foster mothers, women who were pregnant at the time of the interview, and those who were severely ill enough to interfere with the interviewing process were excluded. A total of 485 respondents were recruited within two months period. Using the simple random sampling technique, 5 public healthcare facilities were selected from a sampling frame of 46 public health facilities. The sample size was proportionally allocated to the selected health facilities based on the proportion of women who attend immunization clinics with their infants. Consecutive sampling was used to recruit the respondents until the required sample size was attained.

**Measurement of variables:** the dependent variable was the unmet need for PPFP. This was assessed as ‘yes’ or ‘no’, after evaluating the targeted questions as done in the NDHS questionnaire. The targeted questions included assessing the current use of FP, for limiting or spacing. Those who said “no”, were further evaluated for their intent to use FP either for spacing of birth intervals or limiting family size. The independent variables included socio-demographic characteristics, reproductive characteristics, awareness and knowledge of PPFP. Awareness was assessed with the question, “Are you aware of PPFP?”. Knowledge was assessed with a set of 2 questions, the first question was “ What are the benefits of PPFP?”. Four correct answers were expected, each scoring 1 point. The second question asked, “Select the one(s) you know? The options included; male condoms, injectables, IUCD, oral pills, diaphragm, vasectomy, tubal ligation, spermicides, lactational amenorrhoea method (LAM), implants and emergency contraceptive pills (Postinor 2) got 1 point each. An incorrect response mentioned, or a correct response not mentioned was scored zero. This gave a minimal and maximal score of 0 and 15 points respectively. Scores of 7 or higher were considered good knowledge, while scores less than 7 were considered poor knowledge.

**Sample size determination:** the sample size was calculated using the Lesley Kish (1965) formula for single proportion studies as given by the formula; Z^2^pq/d^2^ [23]. An unmet need for postpartum family planning rate (p) of 59.4% based on a previous study in Southwest Nigeria [24], with a precision of 5%, was used to compute the sample size. A non-response rate of 20% was assumed, giving a minimum sample size of approximately 464 participants.

**Data collection tool and methods:** a semi-structured interviewer-administered questionnaire, adapted and modified from the NDHS [25], was used to collect information from the respondents. The Igbo version created by a lecturer versed in the Igbo language was also available for those who may have wished to respond in their native dialect. The reliability and validity of the questionnaire were assessed using the content and face validity techniques. The questionnaire had four sections. The first section contained the socio-demographic characteristics of the respondents, such as age, marital status, residence, religion, denomination, respondents' and partner' education, respondents' and partners' occupation. The second section addressed the reproductive characteristics, including parity, number of living children, intention to use contraceptives, resumption of menses and sexual activities, number of ANC visits and place of delivery. The third section constituted questions on the awareness and knowledge of (PPFP) uses and methods, while the last section comprised the questions for assessing unmet needs of PPFP and the reasons for its practice. To establish face validity, a pretest was done in a health facility in Aba South (a non-sampled LGA) using 5% of the sample size. The result of the pretest was used to improve the clarity and wording of the questionnaire and the logical sequence of the questions. Efforts were made to ensure that the population of postpartum women to be used for the pretest had similar socio-demographic characteristics to the study population in the study sites.

A total of five research assistants were recruited for this study. They were trained by the principal investigator on the ethics and interviewing process of the research within 2 days period. The proposed respondents approached in the immunization clinic were informed by the officer in charge about the study, and the trained research assistants provided the information needed by the proposed respondents. Those who volunteered to participate were enrolled in the study after written informed consent had been obtained. The estimated time for the interview section was 10 to 15 minutes.

### Operational definitions

**Extended postpartum period:** first 12 months following childbirth.

**Unmet need for family planning:** defined as those who are fecund and sexually active but are not using any method of contraception, and report not wanting any more children or wanting to delay the next child.

**Postpartum family planning:** defined as the prevention of unintended pregnancy and closely spaced pregnancies through the first 12 months following childbirth.

**Modern contraceptives:** a product or medical procedure that prevents reproduction from acts of sexual intercourse. They include female and male sterilization, intrauterine contraceptive devices (IUCD), subdermal implants, injectables, oral contraceptives, condoms (male and female), Emergency contraceptives pills, patches, diaphragm and cervical caps, spermicidal agents (gels, foams, creams, suppositories, vaginal rings, sponges).

**Statistical analysis:** the data were entered into Microsoft Excel and analyzed using SPSS version 26. Data cleaning and coding were done, frequency tables and proportions were generated. Appropriate charts were used for further representation of the data. Bivariate analysis was used to test for associations between the independent variables and unmet needs for PPFP. P values <0.05 were considered significant. Using logistic regression, we determined the predictors of unmet need for PPFP in the extended postpartum period. Factors that fitted into the regression model, were those with P values <0.2 at the level of bivariate analysis and those reported from published literature were added to the model. The adjusted odds ratios and 95% confidence intervals were obtained at a level of significance of 5%.

**Ethical consideration:** ethical clearance was obtained from the Research Ethical Committee of Federal Medical Centre Umuahia Abia State with the reference number FMC/QEH/G.596/Vol.10/567. Respondents' privacy and confidentiality were maintained. Written informed consent was obtained and the respondents were made to understand that their participation was voluntary. Data security was assured by storing the data on a passworded computer only accessible to the principal investigator.

## Results

**Socio-demographic characteristics of respondents:** a total of 470 respondents participated in the study out of 485 recruited, giving a response rate of 96.9%. Most of the respondents (32.8%) were within the age group of 25-29 years. The mean age was 30.3 ± 6.1 years among the participants. Most of the respondents (62.1%) reside in urban areas and majority of them (60.4%) had acquired tertiary education. Christianity was the most practiced religion (95.3%) with 43.4% of all the respondents belonging to the Pentecostal denomination. While most of the respondents (36.8%) were salary earners, 33.8% and 29.4% of the respondents were self-employed and unemployed respectively. Furthermore, 56.2% of the respondents earned less than ₦50,000 monthly. Additionally, 66.4% of their spouses had attained tertiary education and close to half were salary earners (48.3%) and self-employed (49.6%) (Table 1).

**Table 1:** socio-demographic characteristics of respondents (N=470)

Variables	Frequency	Percentage (%)
**Age**		
<25	69	14.7
25-29	154	32.8
30-34	133	28.3
35 - 39	70	14.9
≥40	44	9.4
Mean ± SD	30.3 ± 6.1	
**Residence**		
Rural	178	37.9
Urban	292	62.1
**Educational status**		
≤ Secondary	186	39.6
Tertiary	284	60.4
**Religion**		
Christianity	448	95.3
Others	22	4.7
**Denomination**		
Catholic	134	28.5
Orthodox	110	23.4
Pentecostal	204	43.4
Others	22	4.7
**Employment status**		
Salary earner	173	36.8
Self-employed	159	33.8
Not employed	138	29.4
**Monthly household income (Naira ₦)**		
< ₦50,000	264	56.2
>₦50,000	206	43.8
**Spouses’ educational status**		
Primary	22	4.7
Secondary	136	28.9
Tertiary	312	66.4
**Spouses’ employment status**		
Salary earner	227	48.3
Self-employed	233	49.6
Unemployed	10	2.1

**Reproductive characteristics of respondents:** majority of the respondents (46.6%) intended to have babies after 2 years. More than two-thirds of the respondents (69.1%) agreed that their spouses were the sole decision makers for FP. Three hundred and eighty-nine (82.8%) of the respondents had their delivery in a health facility. Similarly, 89.8% of them attended ANC with 68.3% of the respondents reporting having received FP counseling. Majority (61.1%) had never used any FP method while 58.5% were non-current users of any FP method. The median menses resumption timing was 3 months (IQR: 2-6) and more than half (54.8%) of the respondents had resumed their menses in less than three months after childbirth. A greater proportion of the respondents (89.4%) were HIV-negative (Table 2).

**Table 2:** reproductive characteristics of respondents (N=470)

Variables	Frequency	(%)
**Reproductive intentions**		
Have baby(ies) soon	84	17.9
Have baby(ies) after 2 years	219	46.6
Limit childbirth	83	17.7
Undecided	84	17.9
**Decision making**		
Only me	63	13.4
Jointly with spouse	82	17.4
Only spouse	325	69.1
**Number of living children**		
1	142	30.4
2	143	30.4
3	104	22.1
≥4	80	17.0
Median Parity (IQR)	2 (1,3)	
**Source of delivery**		
Health facility	389	82.8
Home	19	4.0
Traditional birth home	50	10.6
Church	12	2.6
**Attended ANC**		
Yes	422	89.8
No	48	10.2
**FP counseling**		
Yes	321	68.3
No	149	31.7
**Ever used FP**		
Yes	183	38.9
No	287	61.1
**Currently using FP**		
Yes	164	34.9
No	275	58.5
**Menses resumption timing**		
< 3 months	126	54.8
4-6 months	56	24.3
≥ 6 months	48	20.9
Median (IQR)	3(2-6)	
**HIV status**		
Positive	27	5.7
Negative	420	89.4
Not sure	23	4.9

ANC Antenatal care IQR Interquartile range HIV Human Immunodeficiency virus FP family planning

**Figure 1:**
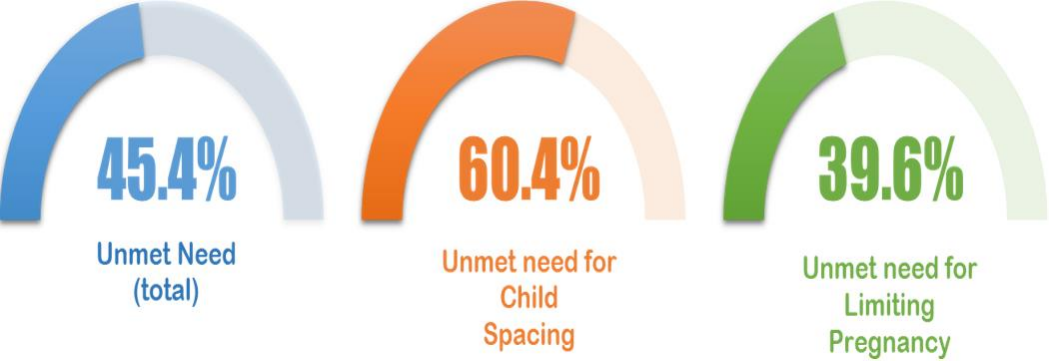
unmet need for family planning among non-FP users

**Prevalence of unmet need for family planning:** the total UMN for FP among non-FP users was 45.4% (95% CI: 40.2-50.7), out of which 60.4% (95% CI: 52.1-68.1) had a UMN for child spacing while 39.6% (95% CI: 31.8-47.8) had UMN for limiting pregnancies. In addition, the met need of PPFP for child spacing was 70.1% (95% CI: 62.7%-76.6%) and 29.9% (95% CI: 23.4%-37.3%) for limiting pregnancies among the respondents (Figure 1).

**Figure 2:**
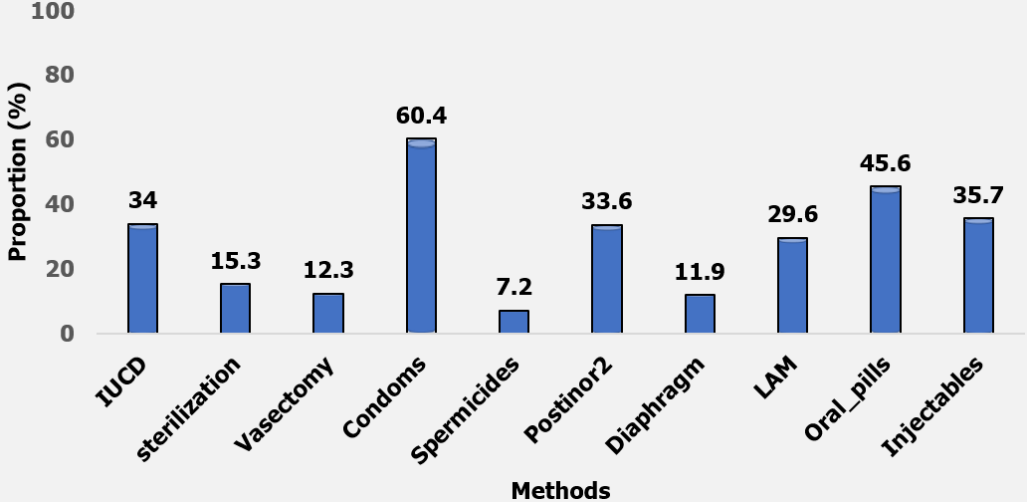
knowledge of post-partum family planning methods

**Knowledge and awareness of postpartum family planning methods:** most of the respondents (60.4%) knew of condoms as a PPFP method. This was followed by oral pills (45.6%), injectables (35.7%), intrauterine contraceptive devices (IUCD 34%), postinor2 (33.6%) and lactational amenorrhoea method (LAM 29.6%) with knowledge of spermicides (7.2%) being the least. The proportion of awareness for PPFP was 72% among the respondents (Figure 2).

**Figure 3:**
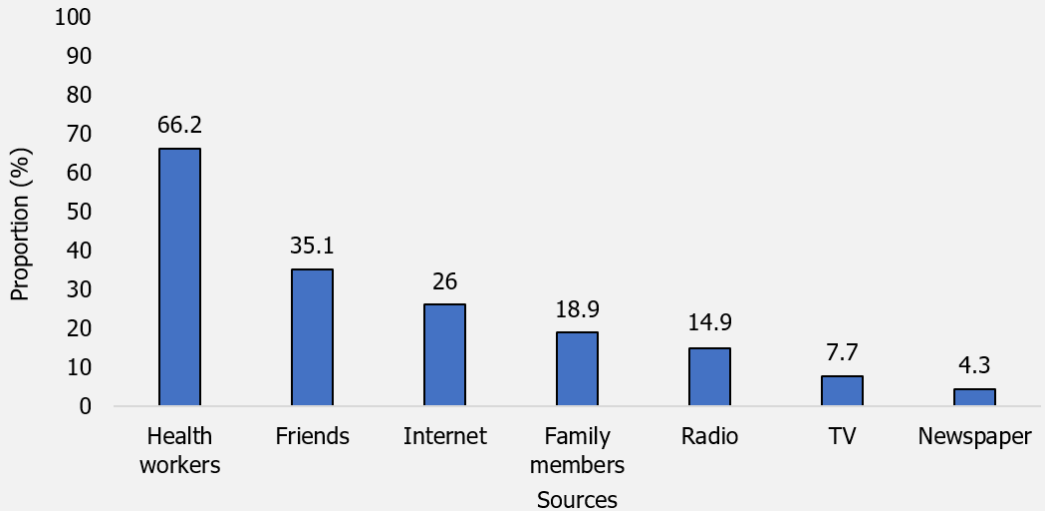
source of information for post-partum family planning methods

**Source of information for postpartum family planning methods:** the most prevalent source of information was from health workers (66.2%). Interestingly, 35.1% of them got their information from friends, while 18.9% of them got theirs from family members. On the other hand, information gotten from TV and newspapers accounted for 7.7% and 4.3%, respectively (Figure 3).

**Figure 4:**
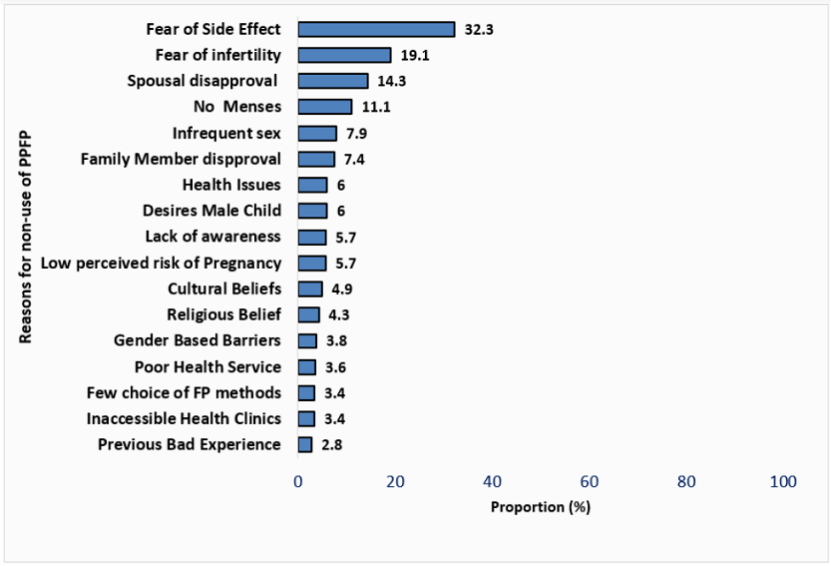
reasons for non-use of PPFP

**Reasons for non-use of postpartum family planning methods:** the commonest reason for non-use of PPFP was the perceived fear of side effects associated with the contraceptives (32.3%). This was followed by the fear of infertility (19.1%). The least prevalent reasons included previous bad experience (2.8%), poor access to clinics and few choices of FP methods available (3.4%) (Figure 4).

**Predictors of unmet need for family planning:** in the bivariate logistic regression model, age, place of residence, educational level, income, knowledge and religion were statistically significantly associated with unmet need for PPFP. Those aged 40 years and above had higher odds of UMN for PPFP when compared to those younger than 40 years (OR = 9.19, 95% CI: 2.94-28.75). Those who resided in rural areas were 67% more likely to have unmet need for PPFP compared to their urban counterparts (OR =1.67, 95% CI: 1.03-2.69). Those who had no formal education and those with primary education were two folds higher to have an unmet need for PPFP compared to those with tertiary education (OR= 2.03, 95% CI: 1.24-3.29). Respondents whose income was less than ₦50,000 had higher odds of unmet need for PPFP when compared to those whose income was above ₦50,000 (OR =3.11, 95% CI: 1.91-5.06). Those with poor knowledge of FP services were more likely to have UMN for FP compared to those who had good knowledge (OR= 12.67, 95% CI: 7.36-21.80). Respondents who were of the Pentecostal denomination had higher odds of UMN for FP (OR= 4.27, 95% CI: 2.28-8.02), as well as those of orthodox denomination (OR =5.51, 95% CI: 2.68-11.31).

After controlling for other variables in the multivariate logistic regression model, the predictors of unmet need for PPFP were; age 35-39 years (aOR=5.39, 95% CI: 1.61-18.06) and ≥ 40 years (aOR= 32.48, 95% CI: 6.48-162.77), rural residence (aOR= 4.27, 95% CI: 2.15-8.47), below tertiary educational level (aOR= 5.21, 95% CI: 2.09-13.01), lower income <₦50,000 (aOR =2.21, 95% CI: 1.10-4.46), poor knowledge of FP (aOR= 33.93, 95% CI: 13.21-87.12), Christians of the Pentecostal denomination (aOR= 4.09, 95% CI: 1.77-9.43) as well as the Orthodox denomination (aOR= 5.44, 95% CI: 2.03-14.58).) (Table 3).

**Table 3:** associated factors of unmet for family planning among women in extended postpartum period

Variable	Unmet need		COR	*P value	aOR (95%CI)	P value
	**Yes**	**No**		ï¿½		
**Age Group**						
<25	16(31.4)	35(68.6)	1		1	
25 - 29	40(44.4)	50(55.6)	1.75(0.85-3.61)	0.129	1.42(0.51-3.97)	0.502
30 - 34	39(42.4)	53(57.6)	1.61(0.78-3.31)	0.196	2.45(0.85-7.07)	0.097
35 - 39	23(48.9)	24(51.1)	2.10(0.92-4.77)	0.078	5.39(1.61-18.06)	0.006
≥40	21(80.8)	5(19.2)	9.19(2.94-28.75)	0.001	32.48(6.48-162.77)	0.001
**Residence**						
Rural	86(67.2)	42(32.8)	1.67(1.03- 2.69)	0.001	4.27(2.15-8.47)	0.001
Urban	53(29.8)	125(70.2)	1		1	
**Educational level**						
Below tertiary	74(55.2)	60(44.8)	2.03(1.24-3.29)	0.002	5.21(2.09-13.01)	0.001
Tertiary	65(37.8)	107(62.2)	1		1	
**Monthly income (Naira ₦)**						
< ₦50,000	103(56.3)	80(43.7)	3.11(1.91-5.06)	0.001	2.21(1.10-4.46)	0.026
>₦50,000	36(29.3)	87(70.7)	1		1	
**Knowledge of PPFP**						
Poor	108(75)	36(25)	12.67(7.36ï¿½21.80)	0.001	33.93(13.21-87.12)	0.001
Good	31(19.1)	131(85.1)	1		1	
**Denomination**						
Catholic	17(21)	64(79)	1		1	
Pentecostal	75(53.2)	66(46.8)	4.27(2.28-8.02)	0.001	4.09(1.77-9.43)	0.001
Orthodox	41(59.4)	28(40.6)	5.51(2.68-11.31)	0.001	5.44(2.03-14.58)	0.001
Others	6(40.0)	9(60.0)	2.51(0.78-8.03)	0.121	1.08(0.24-4.77)	0.922
**Occupation**						
Civil servant	47(42.7)	63(57.3)	1			
Self employed	41(42.3)	56(57.7)	0.96(0.55-1.67)	0.902		
Unemployed	51(51.5)	48(48.5)	1.37(0.79-2.36)	0.255		
**Educational level (Spouse/partner)**				0.123		
Secondary	45(39.5)	69(60.5)	1			
Tertiary	94(67.6)	98(58.7)	1.47(0.89-2.42)	0.123		
**Occupation (Spouse/partner)**				0.391		
Civil servant	66(49.6)	67(50.4)	0.98 (0.23-4.10)	0.999		
Self employed	69(41.8)	96(58.2)	0.71 (0.17-2.97)	0.649		
Unemployed	4(50.0)	4(50.0)	1			

P values <0.05 are considered significant, PPFP Post-partum family planning, COR Crude Odds Ratio aOR Adjusted Odds ratio *binary logistic regression

## Discussion

We conducted this study to determine the prevalence of UMN for PPFP and its predictors among women in the extended postpartum period in Abia State, Nigeria. We found out that one in every two women within the extended postpartum period had an UMN for PPFP. Age, residence, education level, knowledge status and denomination were the predictors of UMN for PPFP. The commonest source of information for FP was the health workers while male condoms were the most common PPFP known by the respondents.

The findings in this study showed that close to half of the respondents had an UMN for PPFP. This contrasts with studies in Nigeria, where higher rates were found in the South-South (97.3%) and Southwest (59.4%) regions [24,26]. Comparatively, lower rates have been reported in Kenya (30%) [19], Ethiopia (36.7%) [27] and Malawi (35%) [16]. However, an earlier study in Ethiopia reported a similar rate of 44% [20]. In Southeastern Nigeria, lower rates of 17.5% to 26.3% were observed among non-postpartum women within the childbearing age (15-49 years) [28-30]. Postpartum women tend to have a low perceived risk of pregnancy. This could be explained in part by the fact that they are breastfeeding, have not resumed menstruation, and have infrequent sexual relations with their spouse.

We found that women in the older age group category had higher odds of UMN when compared to those in the younger age group. This is confirmed by a previous study in West Java [31]. This finding, however, contradicts studies in Nigeria [32], Ethiopia [33,34], sub-Sahara Africa (SSA) based study [35], Mexico [36] and Indonesia [37]. Older women are likely to have less frequent access to health facilities compared to younger ones, where women benefit from FP counseling and other health talks. Our study reported that the commonest sources of information for FP among the respondents were health workers. Older women are not likely to easily adapt to the existing social media platforms, which are also a source of information for FP. They are also likely to have completed their family size but may have some concerns about FP usage. In this study, perceived fear of side effects of the contraceptives was the most common reason for non-use of PPFP. This has been reported in earlier studies [28,31,38].

Education was a significant predictor of UMN in this study. Lower educational status was positively associated with UMN. Other studies in Nigeria, some countries in SSA and Ethiopia have shown similar patterns in the relationship between educational status and UMN [32,35,39]. Female education is paramount to maternal and child health. Educated women are likely to have adequate knowledge of the use of modern contraceptives [40]. An educated woman is likely to be empowered to take part in joint decision-making for FP [40].

Women with low income had higher odds of UMN. This finding was consistent with studies in Nigeria, SSA and West Java [31,32,35]. Inadequate sources of funds tend to reduce the capacity to seek for contraceptives. Their poor income status may also contribute to their inability to acquire televisions and/or radios which are helpful for communication. Additionally, women in the high-income category can plan and limit their pregnancies to avoid interruption to their work schedule.

Poor knowledge of PPFP was positively associated with UMN. This is similar to the findings of studies conducted in Nigeria, Ethiopia and Indonesia, where people with good knowledge of modern contraceptives utilized PPFP [21,37,41,42]. Good knowledge increases the intent to use PPFP. An increase in the knowledge of PPFP leads to a reduced perception of the myths associated with FP utilization. In this study, Orthodox and Pentecostal Christians were more likely to have UMN for PPFP. Religious belief is believed to be a possible hindrance to the uptake of FP [43]. An earlier trend analysis in Ethiopia showed an increase in UMN among Orthodox Christians with a progressive shift to Protestants within a 5-year interval [44]. A plausible explanation may be that women who regularly attend religious activities are less likely to get information regarding FP from health facilities [45].

Respondents who resided in rural areas were more likely to have UMN for PPFP. The finding of this study is in line with studies conducted in Nigeria, Ethiopia, and West Java which reported higher odds of UMN among women who live in rural areas [27,28,31,32]. Women in urban areas have better access to information, education, and health services. Furthermore, people in rural areas may not have access to mass media through which FP jingles and other health education talks are disseminated.

The limitation of this study is that it is a cross-sectional study and relied on self-reported measures. The study was facility-based, which could limit its generalizability. However, random sampling was used to select the respondents. Notwithstanding, the major strength lies in the fact that it is among the first studies to assess UMN among postpartum women following the COVID-19 pandemic. This contributes to measuring the magnitude of the burden of UMN following the pandemic that hindered the utilization of health services due to lockdown measures and overburdened health systems.

## Conclusion

In this study, close to half of the respondents had UMN for PPFP and this was differentially higher for child spacing compared to limiting pregnancies. The independent predictors included older age, lower educational status, low income, poor knowledge, residence and denomination. This study also highlighted the commonest form of PPFP known as male condoms. The most prevalent source of information for FP was from health workers and fear of side effects from the contraceptives was the most common reason for non-use of FP. Family planning interventions should prioritize older women, women living in rural areas with low income, women of lower educational status, including those with poor knowledge of PPFP and who belong to the Pentecostal and Orthodox denominations. Opportunities for intensifying FP counseling should be utilized when women visit health facilities. Furthermore, religious leaders should be educated on FP so that they can pass it on to women in the various worship centres.

### What is known about this topic

- *Unmet needs for family planning accounts for poor maternal and child health indicators;*
- *Women in the postpartum period contribute largely to the proportion of unmet needs of family planning;*
- *The COVID-19 pandemic has affected the utilization of reproductive health services including family planning services in the facilities*.

### What this study adds

- *This study provides an estimate of the unmet needs of FP among women in the extended postpartum period in Abia State, within the COVID-19 pandemic era;*
- *This will guide stakeholders in the design and evaluation of FP programmes;*
- *This study gives insight into the significant predictors of UMN within the context of the study area*.
